# The role of conservative versus innovative nesting behavior on the 25‐year population expansion of an avian predator

**DOI:** 10.1002/ece3.3007

**Published:** 2017-05-04

**Authors:** Andreia Dias, Luís Palma, Filipe Carvalho, Dora Neto, Joan Real, Pedro Beja

**Affiliations:** ^1^ Equip de Biologia de la ConservacióDepartament de Biologia EvolutivEcologia i Ciències Ambientals and Institut de la Recerca de la Biodiversitat (IRBIO)Universitat de BarcelonaBarcelonaCataloniaSpain; ^2^ CIBIO/InBio‐UPCentro de Investigação em Biodiversidade e Recursos GenéticosUniversidade do PortoVairãoPortugal; ^3^ CIBIO/InBIO‐UECentro de Investigação em Biodiversidade e Recursos GenéticosUniversidade de ÉvoraÉvoraPortugal; ^4^ Department of Zoology and EntomologySchool of Biological and Environmental SciencesUniversity of Fort HareAliceSouth Africa; ^5^ CEABN/InBIOCentro de Ecologia Aplicada “Professor Baeta Neves”Instituto Superior de AgronomiaUniversidade de LisboaLisboaPortugal

**Keywords:** *Aquila fasciata*, behavioral innovation, conditional logistic regression, conservation, habitat selection, quantile regression, range expansion

## Abstract

Species ranges often change in relation to multiple environmental and demographic factors. Innovative behaviors may affect these changes by facilitating the use of novel habitats, although this idea has been little explored. Here, we investigate the importance of behavior during range change, using a 25‐year population expansion of Bonelli's eagle in southern Portugal. This unique population is almost exclusively tree nesting, while all other populations in western Europe are predominantly cliff nesting. During 1991–2014, we surveyed nest sites and estimated the year when each breeding territory was established. We approximated the boundaries of 84 territories using Dirichlet tessellation and mapped topography, land cover, and the density of human infrastructures in buffers (250, 500, and 1,000 m) around nest and random sites. We then compared environmental conditions at matching nest and random sites within territories using conditional logistic regression, and used quantile regression to estimate trends in nesting habitats in relation to the year of territory establishment. Most nests (>85%, *n* = 197) were in eucalypts, maritime pines, and cork oaks. Nest sites were farther from the nests of neighboring territories than random points, and they were in areas with higher terrain roughness, lower cover by agricultural and built‐up areas, and lower road and powerline densities. Nesting habitat selection varied little with year of territory establishment, although nesting in eucalypts increased, while cliff nesting and cork oak nesting, and terrain roughness declined. Our results suggest that the observed expansion of Bonelli's eagles was facilitated by the tree nesting behavior, which allowed the colonization of areas without cliffs. However, all but a very few breeding pairs settled in habitats comparable to those of the initial population nucleus, suggesting that after an initial trigger possibly facilitated by tree nesting, the habitat selection remained largely conservative. Overall, our study supports recent calls to incorporate information on behavior for understanding and predicting species range shifts.

## 1 Introduction

The geographic range of species is dynamic, often contracting, expanding, or otherwise changing its limits in relation to multiple environmental and demographic drivers (Gaston, 2003). In general, it is expected that a species range will track changes in the geographic distribution of favorable climates and habitats, under the constraints of dispersal limitation (Robillard, Coristine, Soares, & Kerr, 2015; Schloss, Nuñez, & Lawler, 2012; Sohl, 2014). This view has been used to forecast species range shifts in relation to climate and land use changes (Robillard et al., 2015; Schloss et al., 2012; Sohl, 2014) or to predict the ranges of exotic species introduced into new areas (Peterson, Papes, & Kluza, 2003; Veech, Small, & Baccus, 2011). Implicit within this idea, however, is that climatic and habitat niches are conserved during range shifts, which may not be warranted due for instance to evolutionary adaptations to changing conditions or the emergence of behaviors that facilitate the use of novel habitats (Broennimann et al., 2007; Van Dyck, 2012; Wright, Eberhard, Hobson, Avery, & Russello, 2010). Understanding these processes is essential to predict species responses to environmental changes (Lavergne, Mouquet, Thuiller, & Ronce, 2010).

Behavioral innovations, defined as the ability of animals to invent new behaviors or adjust old behavior to new problems (Overington, Griffin, Sol, & Lefebvre, 2011; Sol, Sayol, Ducatez, & Lefebvre, 2016), may be particularly important during range expansion, when species are bound to face novel environmental conditions (Keith & Bull, 2017). For instance, species colonizing landscapes modified by humans often show behavioral adaptations such as changes in the timing of breeding, adjustments of diel activity patterns, and the use of new food sources and foraging strategies (Lowry, Lill, & Wong, 2013; Martínez‐Abraín & Jiménez, 2016). Innovations in breeding habitats include, for instance, avian nesting in human structures such as houses and electric pylons, which permit overcoming scarcity of natural nesting substrates (Martínez‐Abraín & Jiménez, 2016). Likewise, increasing behavioral tolerance toward humans is normally considered a prerequisite for a species to colonize urban habitats and other heavily disturbed areas (Lowry et al., 2013). Despite these benefits of innovation, however, animal behavior may often be conservative rather than innovative, thereby restricting or at least delaying range expansion into potentially suitable habitats (Keith & Bull, 2017; Sol et al., 2016). For instance, imprinting of young to natal habitat characteristics is judged to strongly constrain breeding habitat selection when individuals reach maturity (Davis & Stamps, 2004). Overall, therefore, it is likely that species colonizing new geographic areas should be strongly affected by conservative versus innovative behaviors, although long‐term studies examining this topic are lacking.

**Figure 1:**
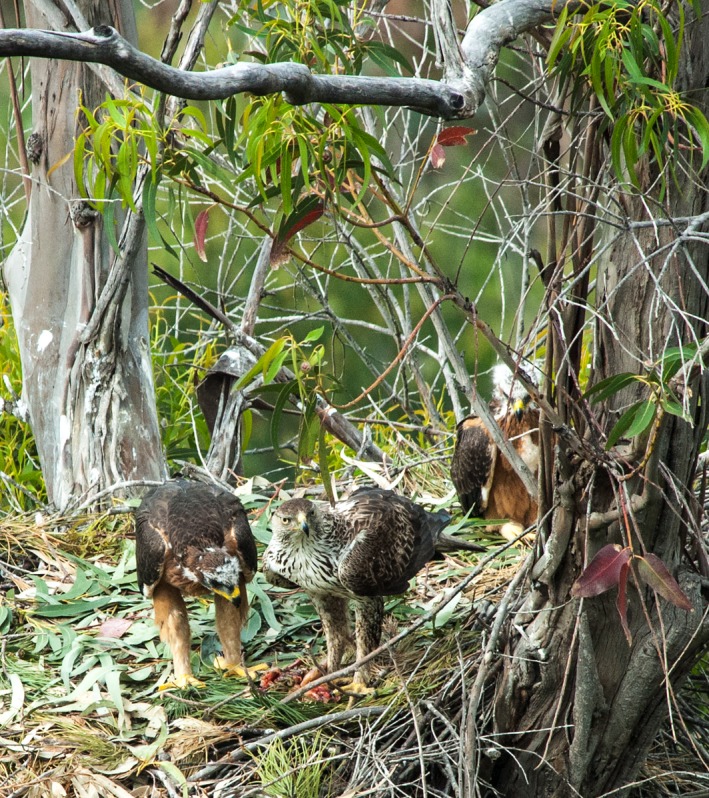
Bonelli's eagle (*Aquila fasciata*) nest in a eucalyptus tree, with one adult and two well grown chicks. Photograph by Joaquim Pedro Ferreira

The Bonelli's eagle (*Aquila fasciata*) in southern Portugal provides a valuable opportunity to examine the role of behavior during a long‐term process of expansion. The Bonelli's eagle is a medium/large bird of prey that is endangered in Europe, where it is largely confined to the Mediterranean region and its numbers have declined since the early 1980s (Hernández‐Matías et al., 2013). In Western Europe, the Bonelli's eagle has a metapopulation‐like structure with a source‐sink dynamics, where the only growing populations are those of southern Spain and southern Portugal (Hernández‐Matías et al., 2013). The population of southern Portugal is peculiar, because it is almost exclusively made up of tree nesting pairs (Figure 1) and is genetically divergent, whereas Bonelli's eagle populations in northern Portugal and elsewhere in the Iberian Peninsula and France are largely dominated by cliff nesters and well‐connected demographically and genetically (Hernández‐Matías et al., 2013; Mira, Arnaud‐Haond, Palma, Cancela, & Beja, 2013; Palma, Beja, & Sánchez, 2013). This population has been closely monitored during the past 25 years, while it grew from about 25 to at least 110 breeding pairs (Beja & Palma, 2008; Palma et al., 2013). The original nucleus was largely confined to the uplands of the extreme south of the country, where the landscape is dominated by forests and scrubland, and human population density is low, while the current population occupies a much larger geographic area with a wide range of habitats and human occupation patterns (Palma et al., 2013). Evidence from demographic modeling and genetics suggests that population growth was sustained by the intrinsic demography of the original nucleus, rather than immigration (Hernández‐Matías et al., 2013; L. Palma and R. Godinho Unpublished Data). Presumably, tree nesting behavior had an important role in this expansion, by allowing new pairs to establish in cliffless areas in a wide range of landscape types (Palma et al., 2013). It is uncertain, however, whether this expansion was associated with innovation in terms of new habitats occupied and increasing tolerance toward humans, or rather it was conservative by largely retaining the characteristics of the original population nucleus in terms of nesting substrate and breeding habitats.

Here, we test these ideas by analyzing nesting habitat selection by the Bonelli's eagle, using territories established in southern Portugal from 1990 to 2014 and that were still active at the end of the study period. Specifically, we: (1) assessed the use of nesting substrates by the expanding population; (2) characterized environmental conditions within territories and around nests in terms of dominant habitat features and human occupation patterns; (3) quantified factors affecting nesting habitat selection within territories using conditional logistic regression; and used quantile regression to estimated trends in (4) nesting habitat conditions and in (5) the predictive ability of habitat selection models in relation to the year of territory occupation. If habitat selection was conservative, we expected that nesting substrates and the habitats around nesting sites should not change in relation to the year of territory establishment. Also, there should be no trends in the predictive ability of the nesting habitat model in relation to territory age, as it was calibrated considering all the territories occupied during the 25‐year study period. In contrast, if behavior was innovative, we would expect the occurrence of changes in some of these descriptors, including temporal trends in the mean nesting habitat characteristics, or temporal increases in the variability of such habitats at the population level. Results were used to discuss the importance of innovative versus conservative behavior for the conservation management of Bonelli's eagles and other species of concern.

## 2 Methods

### 2.1 Study area

The study was carried out in southern Portugal, encompassing an area of about 4 × 10^4 ^km^2^. The climate is Mediterranean, with mean annual temperature of ≈17°C, and mean annual precipitation ranging from ≈500 to ≈1,000 mm (IM/AEMet, 2011). The landscape is dominated by an extensive peneplain (200–450 m a.s.l.) punctuated by residual elevations and bordered on its southern and southwestern ends by low altitude (<900 m a.s.l.) uplands. Land cover is varied, but it includes vast areas occupied by irrigated and rainfed annual crops, permanent crops (e.g. vineyards and olive groves), cork oak (*Quercus suber*) and holm oak (*Quercus rotundifolia*) woodlands and agroforestry systems, Blue gum (*Eucalyptus globulus*) and pine (*Pinus* spp.) plantations, and scrublands of diverse structure and composition. Human density is low throughout much of the area, with most population concentrated along the coast and in urban centers in the hinterland.

### 2.2 Study design

**Figure 2:**
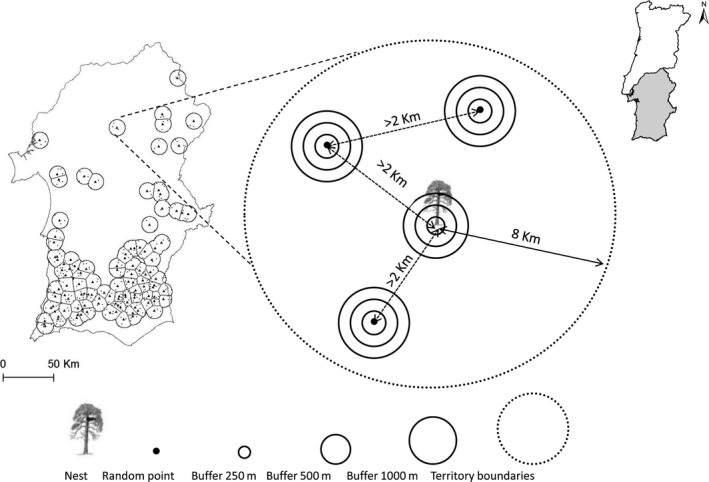
Location of the study area in southern Portugal showing the Bonelli's eagle breeding territories and nests considered in this study (1990–2014), and schematic representation of the study design (see text for details)

Bonelli's eagles are nonmigratory birds of prey, living in pairs that occupy exclusive territories, where there may be one or several alternative nests (Hernández‐Matías et al., 2013; and references therein). The study was based on a long‐term survey (1991–2014) of these breeding pairs and their territories in southern Portugal. For each territory, we estimated the approximate year of first occupation by the breeding pair, and we tried to locate all its nests. In the field, we recorded whether each nest site was built on a cliff or in a tree, and in the latter case, we recorded the nest tree species. Habitats around nests (250‐m, 500‐m and 1,000‐m radius buffers) and random sites (see below) were characterized using variables extracted from GIS layers. We considered three buffers, because factors operating at different spatial scales may affect the selection of nesting habitats. The analysis of habitat selection was based on the comparison of habitat conditions at matching nest and random sites within territories. For each breeding pair, we retained in analysis all nests at >2,000 m from each other, to avoid overlapping buffers. For each group of nests at <2,000 m from each other, we retained the one used most frequently during the study period. Every nest site of each breeding pair was then matched with three points randomly located at >2,000 m from each other and from the nest site, within the corresponding territory boundary (Figure 2). The number of random points was a compromise between the need to avoid overlapping buffers, and to sample adequately the habitat available within each territory (e.g., Carvalho, Carvalho, Mira, & Beja, 2016). To avoid trivial results, random points falling within urban areas and water reservoirs were randomly relocated. To infer eventual behavioral changes during expansion, we estimated temporal trends in the mean and in the variability of nesting habitat conditions.

### 2.3 Bonelli's eagle data

The methods used to collect comprehensive information on the breeding Bonelli's eagle population in southern Portugal have been detailed elsewhere (e.g., Beja & Palma, 2008; Hernández‐Matías et al., 2013; Palma, Beja, Pais, & Cancela da Fonseca, 2006; Palma et al., 2013). Briefly, we conducted surveys throughout the region during courtship, nest building and breeding to locate Bonelli's eagle territories, focusing primarily on areas with potentially suitable habitats. In addition, surveys were directed toward areas with historical information on breeding sites, and areas with observations of individuals reported by other researchers and birdwatchers. Repeated observations of one or two adults or subadults within circumscribed areas were used to identify potentially breeding territories, which were then thoroughly surveyed until nests were found. A breeding territory was considered to be present in a given area when at least one nest was located, and there was at least one breeding attempt (i.e., at least nest building or repair) in at least 1 year. The year of territory establishment was estimated from a combination of information sources, including mainly the history and spatial pattern of Bonelli's eagle observations in the area, and enquiries to key informers such as shepherds and game managers. Frequently, estimates were made in terms of a likely time interval, for which we used the mid‐point in subsequent analysis. Breeding data were collected for the active nests located each year in each territory, based on observations carried out using binoculars and telescopes (20–60×) from a distance to minimize disturbance.

To match nest sites with random locations within the same territory, we defined the territory boundary of each breeding pair as an 8‐km radius buffer around its central point, which was estimated as the geographic centre of all the nests of the breeding pair (Palma et al., 2006). When the centers of neighboring territories were at <16 km from each other, the territory boundaries were defined using Dirichlet tessellation (Schlicht, Valcu, & Kempenaers, 2014). These assumptions were similar to those taken in a previous study where we found a good matching between diets and food resources across territories (Palma et al., 2006), and they were based on home range data from satellite tracking of ten breeding adults in our study area (L. Palma, unpublished data). Therefore, we believe that these territories provided a reasonable approximation to select random points and thus estimate the habitats available to each breeding pair, although it does not account for eventual variations in territory sizes and shapes (e.g., Bosch, Real, Tintó, Zozaya, & Castell, 2010; Mure, 2003).

### 2.4 Environmental variables

The buffers around nest sites and random points were characterized from 15 variables reflecting topography, human disturbance, land cover, and potential intraspecific interactions (Table 1, Table S1), which were expected to influence Bonelli's eagles (e.g., Carrascal & Seoane, 2008; Di Vittorio, Sarà & López‐López, 2012; Muñoz & Real, 2013; Real, Bosch, Tintó, & Hernández‐Matías, 2016). All variables were extracted on a GIS from digital thematic layers, using ArcMap 10.1. Topographic variables were estimated using a 25‐m resolution digital elevation model (http://www.eea.europa.eu/dataand-maps/data/eu-dem). For each buffer, we computed the means and standard deviations of elevation and slope of raster grid cells, and we estimated an index of ruggedness using the Vector Ruggedness Measure Tool (Sappington, Longshore, & Thompson, 2007). This index measures terrain ruggedness as the variation in three‐dimensional orientation of grid cells within a neighborhood, effectively capturing variability in slope and aspect into a single measure (Sappington et al., 2007). The density of paved roads was estimated using the Open Street Map (www.openstreetmap.org/copyright), and it was taken as a broad indicator of potential human disturbance. Distribution power lines were also taken as an indicator of potential disturbance because they are a source of mortality in Bonelli's eagles (Real, Grande, Mañosa, & Sánchez‐Zapata, 2001; Rollan, Real, Bosch, Tintó, & Hernández‐Matías, 2010), and their density was estimated from electric network maps. Land cover was estimated using Portugal's 2007 Land Cover Map with land cover classes aggregated in five main categories judged a priori to be the most relevant for Bonelli's eagles nesting habitat selection (see Table 1 for details). We have used relatively broad habitat land cover classes, because they have changed less over time than more detailed categories (ICNF, 2013), thereby reducing errors potentially associated with considering only a land cover map from 2007 to analyze habitat selection from territories established between 1990 and 2014. We also estimated the density of waterlines, because Bonelli's eagles frequently nest along streams and gullies (Palma et al., 2013). Finally, we considered the distance to the nearest nest of a different breeding territory, to account for the possibility of individuals avoiding sites because of their proximity to those occupied by neighboring breeding pairs.

**Table 1:** Variables used to analyze the environmental correlates of nesting site selection by the Bonelli's eagle in southern Portugal

Variable (unit)	Code	Description (transformation)
Topography		
Elevation (m)	ELMEN	Elevation above sea level (DEM 25 m)—mean and standard deviation (log10)
	ELSTD	
Slope (°)	SLMEN	Slope—mean and standard deviation (log10)
	SLSTD	
Ruggedness Index	VRMEN	Terrain ruggedness measured as the variation in three‐dimensional orientation of grid cells within a neighborhood—mean and standard deviation (log10)
	VRSTD	
Human disturbance		
Paved road network (m/m2)	DEPR	Density of paved roads (Asin [√x])
Power line (m/m2)	DEPL	Density of High/Very High Tension (>60 kv) and Medium Tension (<60 Kv) power lines (Asin [√x])
Land cover		
Artificial areas (%)	EXAR	Proportion of artificial areas (urban areas, industrial, commercial and industrial units, mine, dump and construction sites, artificial nonagricultural vegetated areas) (Asin [√x])
Agricultural areas (%)	EXAG	Proportion of heterogeneous agricultural areas, permanent pastures and crops, arable land and rice fields (Asin[√x])
Forests (%)	EXFO	Proportion of forests (broad leaved forests, coniferous forests, mixed forests) (Asin [√x])
Open forests (%)	EXOF	Proportion of open forests, shrubs, herbaceous vegetation, and open spaces with little or no vegetation (Asin [√x])
Water bodies (%)	EXWA	Proportion of water bodies (e.g., reservoirs, lagoons) and wetlands (Asin [√x])
Waterline (m/m^2^)	DEWL	Density of waterlines (Asin [√x])
Intraspecific relationship		
Distance to nest (m)	DIBN	Distance to the nearest Bonelli's eagle nest (log10)

### 2.5 Data analysis

Prior to statistical analysis, skewed variables were transformed to approach normality and to reduce the influence of extreme values using the angular and logarithmic transformations (Table 1). All variables were standardized to zero mean and unit variance, to enhance comparability of effect sizes (e.g., Schielzeth, 2010). Principal component analyses (PCA) of ecological variables were used to investigate multicollinearity and to describe dominant environmental gradients (Legendre & Legendre, 1998). Varimax normalized rotations were applied to the set of principal components with eigenvalues >1, to obtain simpler and more interpretable gradients (Legendre & Legendre, 1998). Varimax rotated axes were then used in subsequent analysis, because they provide a reduced set of synthetic variables, which are orthogonal to each other and thus are not affected by multicollinearity. A separate PCA and varimax rotation was carried out for variables estimated in 250‐, 500‐, and 1000‐m buffers, because we were interested in modeling habitat selection in relation to scale‐specific factors. We excluded the distance to the nearest nest from PCAs, because we were interested in estimating its unique effect and because preliminary analysis showed that it was uncorrelated with other variables.

The factors influencing nest site selection were analyzed at each spatial scale by comparing nest site and random locations within territories, using conditional logistic regression (Duchesne, Fortin, & Courbin, 2010; Hosmer & Lemeshow, 2000). This analysis followed a match‐control design framework, using a binomial variable coding the nest (1) vs. three random points (0), thereby creating a group “stratum” (e.g., Hosmer & Lemeshow, 2000). Model selection was based on the information theoretical approach of Burnham and Anderson (2002) using the Akaike Information Criterion (AICc) and the corresponding Akaike weights (*w*_i_). Candidate models were built based on all possible subsets of the ecological gradients obtained in the vPCA, including the null (i.e., without explanatory variables) and the full (i.e., with all explanatory variables) models. Models were ranked according to their Akaike weights (*w*_i_), and the average parameters and their unconditional standard errors (*SE*) were estimated based on the 95% confidence set of models (Burnham & Anderson, 2002). The relative importance of each variable (ecological gradient) was judged based on the sum of Akaike weights of models where the variable was included (w_+_), and on the magnitude of the average model coefficient. Model fit was assessed with the pseudo R‐squared of Tjur (2009), and model discrimination ability was assessed with the area under the remote operating characteristic curve (AUC; Fielding & Bell, 1997). These analyses were performed using the packages mclogit (Elff, 2013), MuMIn (Barton, 2013), and modEva (Barbosa, Brown, Jiménez‐Valverde, & Real, 2014) for R 3.3.2 software (R Development Core Team 2016).

The analysis of trends in nesting habitats was based on quantile regression, following the rationale outlined in Cade and Noon (2003). This approach was used because we were interested in assessing changes over time in the mean (median) habitat conditions used by breeding pairs, but also in whether variability in nesting habitats increased over time due for instance to a few pairs settling in unusual habitats. In quantile regression, the latter hypothesis may be tested by examining temporal rates of change in quantiles near the maximum (e.g., 95% quantile) or the minimum (e.g., 5%), response. Increases in variability of habitat conditions may be inferred when the absolute value of the slopes estimated for extreme quantiles is significantly larger than that estimated for the median response. The analyses focused on the relations between nesting habitat characteristics described using the PCA axis and the first year of territory occupation. Also, we estimated relations between the prediction errors of the habitat model and the year of territory establishment, assuming that changes in behavior would lead to temporal changes in the median or in the variability of the prediction errors, or both. Model prediction errors were computed for nest sites as one minus the model predicted probability that a site was a nest site. Analysis was carried out at the level of breeding territories by averaging variables across all nest sites within each territory. Separate analyses were made for the three spatial extents used in this study (i.e., 250, 500, and 1,000 m). We estimated the temporal trend of the response variables using ordinary least squares, and we then estimated trends in the quantiles from 5% to 95% at 5% intervals. For each coefficient of the quantile regression, we computed the 90% confidence intervals based on inverting a quantile rankscore test (Cade & Noon, 2003). We also compare the slopes of the regression coefficients of the 5% and 95% quantiles with those of the median, using an ANOVA function for quantile regression fits based on the Wilcoxon score (Koenker et al., 2016). In trend analysis, all territories estimated to be present before the beginning of the study in 1991 were assigned to 1990 as the year of establishment. These analyses were performed using the package quantreg (Koenker et al., 2016), and results were visualized using ggplot2 (Wickham & Chang, 2016), for R 3.3.2 software (R Development Core Team 2016).

## 3 Results

### 3.1 Bonelli's eagle nests and nesting population

We estimated the approximate boundaries of 84 Bonelli's eagle territories from 197 nests (mean number of nests per territory [±*SD*, range] = 2.3 ± 1.4, 1–8) detected during the study period (Figure 2). The eagle population in southern Portugal before 1991 was estimated at 25 territories (29.8% of the total studied). For the territories established after 1990 (*n* = 59), the mean (±*SD*) estimated year of establishment was 2004 ± 5.7 years (1992–2012). From all the nests recorded, only 11 (5.6%) were on cliffs, whereas the others (*n* = 186) were in *Eucalyptus globulus* (36.5%), *Pinus pinaster* (18.8%), *E. camaldulensis* (15.2%), *Quercus suber* (14.7%), *Pinus radiata* (7.1%), *Pinus pinea* (1.5%), and *Populus nigra* (0.5%). There was a significant tendency (chi‐squared = 4.80, *p *=* *.030) for the initial territories (<1991) having a higher proportion of nests on cliffs (4.1%) than more recent territories (1.6%). There were also significant differences between periods in the species of nest tree used (chi‐squared = 31.4, *p *<* *.001), mainly due to a higher use of *E*. *camaldulensis* (24.6% vs. 2.8%) and a lower use of *Q. suber* (7.0% vs. 29.2%) in the second period.

### 3.2 Nesting habitat selection

At each spatial scale, the principal component analysis with varimax rotation extracted four dominant environmental gradients that accounted for 68%–74% of total variance in the data and that were largely consistent across scales (Table 2). The dominant gradient (PC1; 36%–38% of variance) was largely related to terrain ruggedness, reflecting a joint increase in mean and standard deviation of slope, standard deviation of elevation and index of ruggedness, and a decline in agricultural land cover. The second gradient was related to human infrastructures (PC2; 12%–17%), showing a joint increase in cover by artificial areas, and in paved roads and powerline densities. The third gradient (PC3; 11%) contrasted areas at higher elevation with lowland areas with more waterlines and waterbodies. Finally, the fourth gradient was mostly related to the increase in forest cover (PC4; 8%–9%), showing a contrast between open and closed woodland at the two smaller spatial scales.

**Table 2:** Scores of habitat variables used to characterize nesting habitats of the Bonelli's eagle in southern Portugal, on the axis (PC#) extracted through a principal component analysis (PCAs) with varimax rotation. Separate PCAs were performed for variables extracted at three spatial scales. We provide the proportion of variance accounted for by each axis extracted in each PCA

Variables	250 m				500 m				1,000 m			
	PC1	PC2	PC3	PC4	PC1	PC2	PC3	PC4	PC1	PC2	PC3	PC4
Mean slope	0.95				0.96				0.96			
Standard deviation of slope	0.95				0.96				0.97			
Ruggedness	0.94				0.94				0.95			
Standard deviation of ruggedness	0.90				0.91				0.92			
Standard deviation of elevation	0.90				0.91				0.90			
Agricultural areas	−0.75				−0.75				−0,79			
Open forests				−0.81	0.55			−0.77	0.59			
Paved road density		0.81				0.84				0.90		
Artificial areas		0.74				0.74				0.84		
Power line density		0.60				0.78				0.83		
Mean elevation			0.73				0.74				0.71	
Waterline density			−0.54				−0.51				−0.56	
Water bodies			−0.81				−0.83				−0.85	
Forests				0.76				0.77				0.78
% Explained variance	36	12	11	9	37	14	11	9	38	17	11	8

The model selection and averaging procedure yielded conditional logistic regression models that were very similar at the three spatial scales considered, consistently showing that within territory boundaries the Bonelli's eagle nests were located in areas with higher terrain ruggedness and lower agricultural cover (PC1), and less human infrastructures (PC2) than random sites (Tables 3 and S2). Also, nests were farther than random points from the nearest nest of a neighbor territory. Support for the negative effect of elevation (PC3) and the positive effect of forest cover (PC4) was moderate at the 1,000‐m scale (Akaike weights > 0.8), but it was weak at lower scales. The T‐Jur coefficients showed that the models at the three spatial scales had a reasonable fit to the data (0.635–0.655), while the AUCs indicated high model discrimination ability (0.946–0.955).

**Table 3:** Average models describing the estimated effects of explanatory variables on the nesting area selection of tree nesting Bonelli′s eagle at three spatial scales: 250, 500, and 1,000 m. For each case, multimodel averaging was based on the 95% confidence set of models. For each variable, we show the standardized regression coefficient (β), the unconditional standard errors (*SE*), the 95% confidence interval of coefficient estimate (CI), and the selection probability (w+). Coefficient estimates whose 95% CI exclude zero are in bold

Variables	β	*SE*	CI	ω+
Buffer: 250 m				
Terrain ruggedness (PC1)	**2.199**	**0.640**	**0.944, 3.455**	**1.000**
Human infrastructures (PC2)	−**3.845**	**1.555**	−**6.893,** −**0.797**	**1.000**
Elevation (PC3)	−0.707	0.533	−1.752, 0.337	0.490
Forests (PC4)	0.529	0.533	−0.516, 1.575	0.380
Distance to nest	**4.626**	**1.157**	**2.357, 6.895**	**1.000**
Buffer: 500 m				
Terrain ruggedness (PC1)	**1.782**	**0.599**	**0.606, 2.957**	**1.000**
Human infrastructures (PC2)	−**1,495**	**0.629**	−**2.728,** −**0.261**	**1.000**
Elevation (PC3)	−0.891	0.458	−1.789, 0.006	0.670
Forests (PC4)	0.607	0.454	−0.283, 1.49	0.490
Distance to nest	**4.336**	**1.088**	**2.203, 6.469**	**1.000**
Buffer: 1,000 m				
Terrain ruggedness (PC1)	**2.550**	**0.965**	**0.659, 4.442**	**1.000**
Human infrastructures (PC2)	−1.833	0.956	−3.709, 0.041	1.000
Elevation (PC3)	−1.143	0.592	−2.304, 0.017	0.800
Forests (PC4)	1.153	0.600	−0.023, 2.330	0.890
Distance to nest	**5.240**	**1.480**	**2.338, 8.142**	**1.000**

### 3.3 Temporal trends

**Figure 3:**
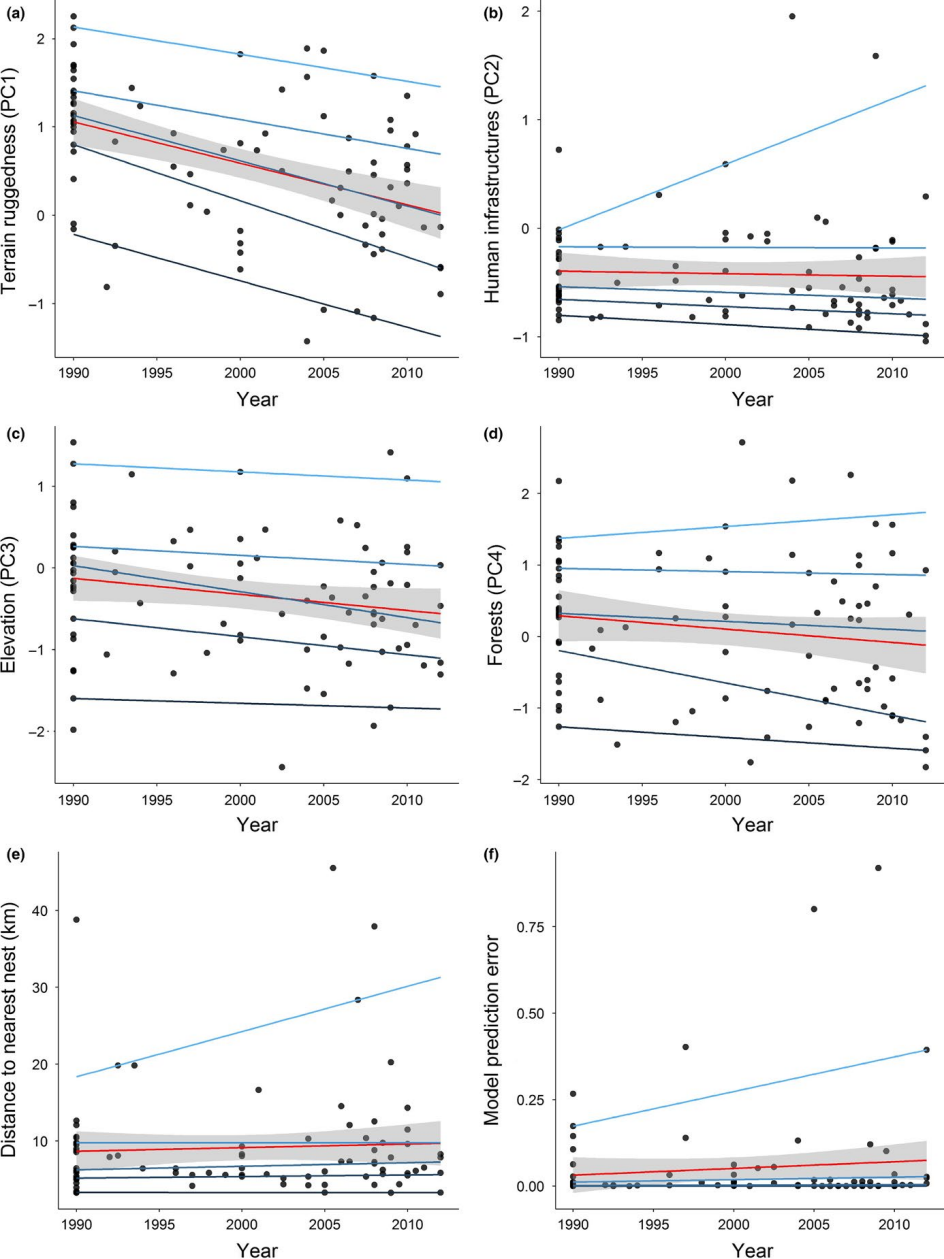
Scatterplots showing trends in habitat conditions around Bonelli’s eagle nests (500‐m buffer) in relation to the time of territory establishment. Trends were estimated using ordinary least squares regression (red line, confidence intervals in gray) and quantile regression (light blue to dark blue lines), considering the habitat gradients extracted from a principal component analysis (PC1‐4; a‐d)), the distances to the nearest nest from a neighboring territory (e), and the prediction error of the habitat model (f). The quantiles represented are 5% (dark blue), 25%, 50%, 75%, and 95% (light blue)

Considering the variables most related to nesting site selection (Table 3), there was a very marked tendency for mean and median terrain ruggedness (PC1) to decline in relation to the estimated year of territory occupation at all spatial scales (Table 4). A similar trend was found for most quantiles at all spatial scales, with no significant differences among slopes (ANOVA, *p *>* *.05), thus suggesting that variability in ruggedness among territories did not change over time (Figures 3 and S1–S5). In contrast, there was no trend in the mean amount of human infrastructures around nests (PC2) in relation to the year of territory establishment, although the median significantly declined at the 250‐m scale (Table 4, Figures 3 and S1–S5). There was also some evidence for increasing variability in more recent territories, as underlined by the contrast between the negative slopes estimated for the lower quantiles (5% and 25%) and the positive slope for the upper quantile (95%), particularly at the 1,000‐m scale. It should be noted, however, that variation among slopes was not significant (ANOVA, *p *>* *.05) and that the response for the 95% quantile appeared driven by a few recent territories with an unusually high amount of human infrastructures around nests (Figures 3, S1 and S2). Regarding the distance to the nearest nest of a neighbor territory, there were no significant trends in the mean or in any quantile, although there were a few recent territories where nests were unusually distant from their nearest neighbors (Table 4, Figures 3 and S1–S5). Considering the less influential variables, there was a tendency for the mean and median (except at 250‐m scale) elevation (PC3), and the median (only at the 1,000‐m scale) of forest cover (PC4), declining in more recent territories, with no significant differences (ANOVA, *p *>* *.05) among the slopes of different quantiles. There was also no evidence for model prediction error varying in relation to the year of territory establishment (Table 4). It is noteworthy, however, that the highest prediction errors were found in a few recent territories (Figures 3, S1 and S2).

**Table 4:** Trends in habitats conditions around Bonelli's eagle nesting sites (250‐, 500‐, and 1,000‐m buffers) in relation to the year of territory establishment. Trends were estimated with both ordinary least squares regression (Mean) and quantile regression (Quantiles), considering the habitat gradients extracted from a principal component analysis (PC#), the distances to the nearest nest from a neighboring territory, and the prediction error of the habitat model. In each case, we provide the slope of the relation, and its 90% confidence interval. Coefficients with confidence interval excluding zero are in bold

Buffer	Mean	Quantiles				
		5%	25%	50%	75%	95%
Terrain ruggedness (PC1)						
250 m	−**0.044 (**−**0.062,** −**0.026)**	−0.047 (−0.087, 0.002)	−**0.061 (**−**0.082,** −**0.017)**	−**0.056 (**−**0.065,** −**0.035)**	−**0.023 (**−**0.051,** −**0.021)**	−**0.046 (**−**0.061,** −**0.009)**
500 m	−**0.047 (**−**0.064,** −**0.030)**	−0.053 (−0.073, 0.002)	−**0.064 (**−**0.081,** −**0.026)**	−**0.052 (**−**0.068,** −**0.035)**	−**0.033 (**−**0.058,** −**0.02)**	−0.031 (−0.062, 0.008)
1,000 m	−**0.046 (**−**0.063,** −**0.029)**	−**0.053 (**−**0.079,** −**0.006)**	−**0.062 (**−**0.079,** −**0.03)**	−**0.052 (**−**0.066,** −**0.025)**	−**0.044 (**−**0.059,** −**0.024)**	−0.025 (−0.059, 5.4 × 10^−5^)
Human infrastructures (PC2)						
250 m	−0.004 (−0.010, 0.002)	−**0.009 (**−**0.013,** −**0.005)**	−**0.010 (**−**0.017,** −**0.005)**	−**0.005 (**−**0.009,** −**2.3** × **10** ^−**4**^ **)**	−0.003 (−0.007, 0.002)	0.012 (−0.013, 0.024)
500 m	−0.002 (−0.013, 0.009)	−**0.009 (**−**0.011,** −**0.005)**	−**0.007 (**−**0.012,** −**0.004)**	−0.006 (−0.017, 3.3 × 10^−5^)	−0.001 (−0.021, 0.010)	0.060 (−0.048, 0.087)
1,000 m	0.005 (−0.005, 0.015)	0.004 (−0.008, 0.005)	−0.003 (−0.011, 0.003)	−0.004 (−0.010, 0.005)	0.002 (−0.006, 0.021)	**0.016 (0.011, 0.084)**
Elevation (PC3)						
250 m	−**0.024 (**−**0.044,** −**0.004)**	−0.010 (−0.048, 0.014)	−0.037 (−0.060, 0.001)	−0.034 (−0.047, 0.011)	−0.021 (−0.04, 0.004)	−0.007 (−0.039, 0.035)
500 m	−**0.020 (**−**0.037,** −**0.002)**	−0.006 (−0.038, 0.031)	−0.023 (−0.047, 0.010)	−**0.032 (**−**0.040,** −**0.009)**	−**0.011 (**−**0.040,** −**0.003)**	−0.010 (−0.073, 0.029)
1,000 m	−**0.023 (**−**0.042,** −**0.005)**	−0.004 (−0.024, 0.047)	−**0.028 (**−**0.057,** −**0.004)**	−**0.030 (**−**0.047,** −**0.006)**	−**0.018 (**−**0.032,** −**0.008)**	−0.039 (−0.066, 0.019)
Forests (PC4)						
250 m	−0.010 (−0.012, 0.032)	−0.013 (−0.026, 0.007)	−0.019 (−0.040, 0.004)	−0.023 (−0.056, 0.019)	−0.008 (−0.029, 0.01)	0.023 (−0.095, 0.088)
500 m	−0.019 (−0.042, 0.004)	−0.015 (−0.039, 0.002)	−0.046 (−0.056, 0.003)	−0.012 (−0.059, 0.003)	−0.005 (−0.039, 0.018)	0.016 (−0.107, 0.059)
1,000 m	−0.022 (−0.046, 0.002)	−0.020 (−0.046, 0.005)	−**0.040 (**−**0.067,** −**0.027)**	−0.04 (−0.056, 0.015)	−0.002 (−0.041, 0.028)	−0.016 (−0.060, 0.052)
Distance to nest						
Distance	47.5 (−120.9, 215.9)	−1.4 (−30.7, 55.9)	20.1 (−75.5, 64.3)	46.1 (−114.7, 89.3)	0.0 (−98.4, 119.8)	588.4 (−986.2, 2110.1)
Model prediction error						
250 m	0.001 (−0.001, 0.003)	0.0 (−1.4 × 10^−8^, 1.2 × 10^−8^)	8.0 × 10^−7^ (−3.0 × 10^−7^, 4.8 × 10^−6^)	4.8 × 10^−6^ (−1.0 × 10^−4^, 5.4 × 10^−5^)	4.8 × 10^−5^ (−9.9 × 10^−4^, 1.2 × 10^−3^)	0.008 (−0.004, 0.030)
500 m	0.002 (−0.001, 0.005)	−7.6 × 10^−8^ (−1.1 × 10^−4^, 9.5 × 10^−8^)	−7.1 × 10^−6^ (−1.0 × 10^−5^, 1.3 × 10^−5^)	1.3 × 10^−4^ (−5.4 × 10^−5^, 5.3 × 10^−4^)	7.2 × 10^−4^ (−0.003, 0.004)	0.010 (−0.021, 0.042)
1,000 m	0.003 (−0.0001, 0.007)	0.0 (−2.5 × 10^−8^, 6.9 × 10^−9^)	5.4 × 10^−7^ (−7.3 × 10^−7^, 3.7 × 10^−6^)	9.1 × 10^−5^ (−3.0 × 10^−5^, 3.1 × 10^−4^)	1.2 × 10^−3^ (4.8 × 10^−4^, 2.1 × 10^−3^)	0.030 (−0.026, 0.051)

## 4 Discussion

Our results suggest that during the 25‐year expansion of Bonelli's eagles in southern Portugal, the nesting habitat characteristics and selection patterns remained very similar to those of the initial population nucleus, albeit with some changes over time. In terms of nest substrate, the tree nesting behavior typical of the initial nucleus was not only retained but even amplified over time, although with some variation in the trees most used. In terms of habitats, nests were consistently located in areas with relatively high terrain ruggedness, low cover by both agricultural land and human infrastructures, and away from conspecific nests in neighboring territories. The main temporal change was a decline in terrain ruggedness around nests in more recent territories, although they were still located within the most rugged areas available within each territory. Mean cover by human infrastructures was little affected by territory age, although variability appeared to be somewhat higher in more recent territories, particularly due to the presence of a few territories with unusually high infrastructure cover around nests. Also, a few recent territories appeared to have an unusual pattern of nesting site selection, as suggested by particularly high model prediction errors. Overall, these results suggest that an initial uncommon behavior, tree nesting, may have triggered the ability of this Bonelli's eagle population to colonize vast areas without suitable cliffs for nesting. However, during the subsequent population expansion, the selection of habitats appeared to be dominantly conservative rather than innovative, although rare unusual behaviors may have started to emerge in recent years.

The interpretation of our results requires due consideration of study design and data analysis approaches, which differed to some extent from other studies on Bonelli's eagle habitat selection. First, our study was conducted at relatively detailed spatial scales, with variables measured at most within 1 km from nests, whereas several other studies considered larger buffers (e.g., Carrete, Sánchez‐Zapata, Martínez, Sánchez, & Calvo, 2002; Di Vittorio, Sara, & López‐López, 2012; Gil Sánchez, Molino Garrido, & Valenzuela Serrano, 1996) or evaluated species presence/absence using 10 × 10 km squares (e.g., Carrascal & Seoane, 2008; Di Vittorio et al., 2012; Muñoz, Márquez, & Real, 2013; Real et al., 2016). This is important because different aspects of Bonelli's eagle habitat selection may become apparent at different spatial scales (López‐López, García‐Ripollés, Aguilar, García‐López, & Verdejo, 2006; Muñoz & Real, 2013; Real et al., 2016), with studies at smaller scales such as ours probably showing the requirements associated with nest sites, and studies at larger spatial scales probably revealing a combination of nesting and foraging habitat requirements. Second, our analysis was based on conditional logistic regression, matching nesting site conditions with those available within territories, whereas all other studies used unmatched comparisons between sites with and without Bonelli's eagles. This may affect results, because conditional regression identifies what is selected considering local availability, and so it is able to reveal selection patterns that might be difficult to discern otherwise (e.g., Carvalho et al., 2016; Duchesne et al., 2010). Finally, our study introduced a temporal dimension to habitat selection patterns that had never been considered before. Although we could not incorporate actual temporal changes in habitat composition due to lack of detailed data, we believe that our approach based on comparisons of current conditions in relation to the year of territory establishment provided a first approximation to how nesting habitat characteristics and selection patterns changed over time. We believe this assumption is reasonable, because the main variables used to characterize Bonelli's eagle habitats have either remained unchanged (e.g., elevation, ruggedness), or they likely varied little over time. In particular, the area occupied by the broad land cover categories used in our study has remained largely stable within Bonelli's eagle territories, as for instance the growth in urban areas and associated infrastructures has been mostly concentrated in a narrow fringe along the coast (Freire, Santos, & Tenedório, 2009), while cover by agriculture and forest areas has remained essentially constant in rural areas of southern Portugal (Godinho et al., 2016; ICNF, 2013). Estimates of the year of territory establishment were associated with some uncertainties, which may have introduced noise in the data but we believe this is unlikely to have biased trends in selection patterns relative to territory age.

Reasons for the association of Bonelli's eagle nesting sites to the most rugged areas within territories may be related to the presence of suitable nesting trees and to less human disturbance (Palma et al., 2013; Real et al., 2016). For instance, large eucalypts are among the most used nest trees and they are most often found along waterlines at the bottom of valleys (Palma et al., 2013), which may be one of the factors attracting the eagles to rougher terrain. Also, rugged areas are probably less affected by forest management operations such as understory clearing for fire prevention (Real et al., 2016; Santana, Porto, Reino, & Beja, 2011) and they may be less often crossed by people. Whatever the reasons for the observed pattern, it is noteworthy that breeding habitat selection of tree nesting Bonelli's eagles in Cyprus was also affected by local topography and the availability of suitable nesting trees away from disturbance (Kassinis, 2010). Cliff nesting Bonelli's eagles also seem to prefer areas with high terrain ruggedness, which seems to reflect the availability of suitable cliffs for nesting (Di Vittorio et al., 2012; Gil Sánchez et al., 1996; López‐López et al., 2006; Real et al., 2016). Overall, therefore, the preference for nesting in rugged areas may be a conservative characteristic of Bonelli's eagles seemingly maintained across geographical regions and nest site typologies, and that may constrain range expansion into milder terrain.

Bonelli's eagle nests were also associated with areas with the lowest cover by built‐up areas and the lowest densities of roads and powerlines. Comparable patterns have been reported elsewhere (Gil Sánchez et al., 1996; López‐López et al., 2006; Real et al., 2016), although other studies did not find significant avoidance of human infrastructures close (<3 km) to occupied nests (Ontiveros 1999; Carrete et al., 2002). Interestingly, Ontiveros (1999) reported that occupied cliffs closer to roads were taller than those farther from roads, suggesting that tolerance to human disturbance may depend on the relative safety of nesting sites (Real et al., 2016; Rollan et al., 2010). Overall, we suggest that our observations regarding human infrastructures, together with the preference for particularly rough terrain, indicates that Bonelli's eagles avoid human disturbance at small distances (<1 km) from nesting sites. It should be noted, however, that our inferences based on conditional logistic regression imply that Bonelli's eagles select the least disturbed areas within their territories, although this may correspond to areas that may still have some human disturbance. Therefore, our results do not contradict the general view that Bonelli's eagles can tolerate a certain degree of human disturbance and that human infrastructures and other indicators of disturbance may be relatively unimportant to explain the species distribution at larger spatial scales (López‐López et al., 2006; Carrascal & Seoane, 2008; Di Vittorio et al., 2012; Muñoz et al., 2013; but see Bosch et al., 2010; Muñoz & Real, 2013 and Real et al., 2016). In addition, it should be noted that a few recent territories had an unusually high cover by human infrastructures around nests, although this patterns was not statistically significant probably due to small sample sizes. The presence of these few pairs apparently more tolerant to human disturbance may imply that in the future the species may be able to expand into more anthropic areas, and this should be the subject of further research.

The trends in nesting habitats in relation to territory age observed in our study suggest that new Bonelli's eagle pairs chose habitats that are structurally comparable to those of the initial population nucleus. This may be a consequence of imprinting of young to natal habitat conditions, which may affect the kind of places the individuals select later in life (Davis & Stamps, 2004). Testing this idea, however, would imply detailed information on the natal and breeding habitats of a large number of marked individuals (e.g., Mannan, Mannan, Schmidt, Estes‐Zumpf, & Boal, 2007), which was unavailable in our case. Nevertheless, there is evidence that the new pairs largely originated from the initial population nucleus, based on the assignment of individuals to the unique genetic profile of the population inhabiting southern Portugal (Mira et al., 2013; Palma et al., 2013), and by the tracking of individuals with conventional and genetic tags (L. Palma and R. Godinho, unpublished). Despite this general trend for conservative behavior, there was still some flexibility in the selection of the nesting area. This was supported to some extent by the decrease in terrain ruggedness in more recent territories, although nests were consistently located in the roughest areas available within territories. Also, there were a few recent territories where nesting site selection was different, as suggested by the higher cover by human infrastructures and the poor predictive ability of the habitat model to differentiate nesting from random sites. Therefore, an even longer time frame would probably be needed to understand whether innovative habitat selection patterns might eventually emerge, although this was not apparent during our 25‐year study.

Taken together, our results suggest that Bonelli's eagles expanded in southern Portugal because the individuals produced by the original nucleus could find vacant nesting habitats of basically similar structure in various landscape types across the region (Beja & Palma, 2008; Palma et al., 2013), rather than through the occupation of novel habitats. Agricultural land abandonment and the depopulation of the countryside since the 1960s was probably responsible to at least some extent for this process, because it released large areas with low disturbance and that have been progressively occupied by uncultivated woodland and scrublands (Diogo & Koomen, 2012; Van Doorn & Bakker, 2007), thus becoming available for Bonelli's eagle colonization during the study period. Another main driver was probably the prevalence of tree nesting behavior, which allowed the colonization of cliffless landscapes that would be unavailable if strict cliff nesting behavior would be retained, as it is commonest in remaining Iberia (Hernández‐Matías et al., 2013; Palma et al., 2013). This idea was supported by genetic studies and demographic modeling, which showed that the genetically isolated tree nesting population of southern Portugal was likely the main source of colonists throughout the expansion process (Hernández‐Matías et al., 2013; Mira et al., 2013; L. Palma and R. Godinho Unpublished Data). Therefore, the conservation of populations with tree nesting behavior may be particularly relevant for the conservation of Bonelli's eagles at wider scales, as this behavioral trait may help the species respond better to ongoing climatic and land use changes (Hernández‐Matías et al., 2013; Muñoz et al., 2013; Palma et al., 2013).

In general, our study shows the importance of understanding the contribution of habitat selection patterns to population expansion (Butcher et al., 2014; Veech et al., 2011). In particular, we showed that species can expand despite a relatively conservative nest site selection behavior, when changes in land use and human demographics provide new vacant areas open to colonization by the growing population (e.g., Balbontin, Negro, Sarasola, Ferrero, & Rivera, 2008; Cardador, Carrete, & Mañosa, 2011). We also found that the fast expansion of this particular eagle population was facilitated by a specific but relatively rare behavior in the Mediterranean region (tree nesting), which allowed the colonization of habitats that otherwise would be unavailable. The study thus adds to the increasing evidence suggesting that preserving behavioral diversity within populations may be essential for species persistence under anthropogenic environmental change (Caro & Sherman, 2012; Sutherland, 1998; Van Dyck, 2012).

## Conflict of Interest

None declared.
