# Supplementary material for: The role of conservative versus innovative nesting behavior on the 25‐year population expansion of an avian predator
Source: Ecol Evol. 2017 May 4;7(12):4241–53. doi: 10.1002/ece3.3007 (PMC5478073; doi:10.1002/ece3.3007)
Supplement: Supplementary file 1 [file ECE3-7-4241-s001.docx]

**SUPPORTING INFORMATION**

**The role of conservative versus innovative nesting behaviour on the 25-year population expansion of an avian predator**

Andreia Dias, Luís Palma, Filipe Carvalho, Dora Neto, Joan Real, Pedro Beja

**Table S1.** Summary statistics (mean ± SD: Range) of habitat variables used to characterise nesting habitat selection by Bonelli’s eagles in southern Portugal. Each variable was estimated around nesting sites and random points, within 250-, 500-, and 1000-m buffers.

| **Variables (code)** | 250 meters | | 500 meters | | 1000 meters | |
| --- | --- | --- | --- | --- | --- | --- |
|  | Nest | Random | Nest | Random | Nest | Random |
| Mean Elevation (m) | 195.9 ± 116.3  (21.5-804.2) | 199.5 ± 111.8  (0.0-722.4) | 197.8 ± 113.8  (21.6-783.0) | 199.6 ± 111.5  (0.0-739.2) | 200.2 ± 108.8  (20.9-729.1) | 200.0 ± 111.1  (0.0-757.0) |
| Standard Deviation of Elevation (m) | 17.3 ± 8.9  (1.8-49.8) | 10.4 ± 8.2  (0.0-48.8) | 23.7 ± 11.8  (3.3-69.1) | 15.7 ± 11.5  (0.0-81.6) | 29.9 ± 14.1  (5.3-76.5) | 21.9 ± 14.5  (0.0-86.3) |
| Mean Slope (°) | 10.1 ± 4.3  (1.3-22.7) | 6.0 ± 4.1  (0.0-20.8) | 9.6 ± 3.9  (1.4-18.8) | 6.0 ± 3.8  (0.0-17.4) | 8.9 ± 3.6  (1.4-17.6) | 6.0 ± 3.5  (0.0-17.1) |
| Standard deviation of slope (°) | 4.7 ± 1.8  (0.7-9.0) | 2.9 ± 1.8  (0.0-9.1) | 4.9 ± 1.7  (0.7-8.5) | 3.3 ± 1.9  (0.0-8.3) | 5.0 ± 1.6  (0.9-8.8) | 3.5 ± 1.8  (0.0-8.3) |
| Mean Ruggedness Index (x10^3^) | 2.6 ± 1.7  (0.1-7.1) | 1.2 ± 0.00130  (0.0-7.3) | 2.4 ± 1.4  (0.1-6.2) | 1.2 ± 01.2  (0.0-5.9) | 2.2 ± 1.3  (0.1-5.7) | 1.2 ± 1.2  (0.0-5.6) |
| Standard Deviation Ruggedness Index (x10^3^) | 1.9 ± 1.2  (0.04-8.0) | 0.9 ± 0.9  (0.0-0.5) | 2.0 ± 1.1  (0.04-5.4) | 1.1 ± 1.0  (0.0-5.7) | 2.0 ± 1.1  (0.2-4.5) | 1.2 ± 1.0  (0.0-4.5) |
| Density of waterlines (m/m^2^;10^3^) | 1.8 ± 6.9  (0.0-6.9) | 1.1 ± 1.6  (0.0-6.2) | 1.6 ± 1.1  (0.0-4.1) | 1.1 ± 1.0  (0.0-4.0) | 1.3 ± 0.6  (0.2-2.7) | 1.0 ± 0.5  (0.0-2.8) |
| Density of paved roads (m/m^2^;10^3^) | 0.0  (0.0-0.0) | 0.4 ± 1.0  (0.0-5.4) | 0.1 ± 0.3  (0.0-2.0) | 0.5 ± 1.0  (0.0-7.1) | 0.1 ± 2.8  (0.0-1.3) | 0.6 ± 0.9  (0.0-7.8) |
| Density of power lines (m/m^2^;10^3^) | 0.1 ± 0.3  (0.0-2.4) | 0.1 ± 1.2  (0.0-7.7) | 0.1 ± 0.3  (0.0-1.5) | 0.6 ± 1.0  (0.0-10.2) | 0.2 ± 0.3  (0.0-1.1) | 0.6 ± 0.8  (0.0-8.5) |
| Proportion of artificial areas (%) | 0.02 ± 0.3  (0.0-2.8) | 0.04 ± 0.2  (0.0-29.4) | 0.2 ± 1.4  (0.0-11.9) | 0.8 ± 3.2  (0.0-37.2) | 0.2 ± 7.8  (0.0-5.8) | 1.0 ± 3.1  (0.0-32.2) |
| Proportion of agricultural areas (%) | 10.0 ± 19.2  (0.0-96.3) | 39.8 ± 40.2  (0.0-100.0) | 13.4 ± 20.2  (0.0-95.8) | 39.3 ± 36.5  (0.0-100) | 16.3 ± 20.5  (0.0-92.6) | 0.386 ± 0.332  (0.000-1.000) |
| Proportion of forests (%) | 29.6 ± 31.7  (0.0-100.0) | 16.9 ± 27.6  (0.0-100.0) | 26.8 ± 28.2  (0.0-98.3) | 16.3 ± 23.9  (0.0-100.0) | 25.7 ± 24.9  (0.0-91.6) | 15.9 ± 20.6  (0.0-95.3) |
| Proportion of open forests (%) | 57.8 ± 34.3  (0.0-100.0) | 41.1 ± 38.6  (0.0-100) | 57.8 ± 30.7  (0.0-100) | 41.9 ± 34.6  (0.0-100) | 56.2 ± 28.3  (0.0-100.0) | 42.6 ± 31.2  (0.0-100.0) |
| Proportion of water bodies (%) | 2.6 ± 5.3  (0.0-25.2) | 1.7 ± 9.0  (0.0-100) | 1.7 ± 3.2  (0.0-14.2) | 1.6 ± 8.3  (0.0-95.4) | 1.6 ± 2.7  (0.0-17.3) | 1.6 ± 7.2  (0.0-0.86.4) |
| Distance to the nearest Bonelli’s eagle nest (m) | 8767.6 ± 6602.6 (3270.3-45507.9) | 3501 ± 1641  (1256-10453) | 8768 ± 6603  (3270-45508) | 3501 ± 1641  (1256-10453) | 8768 ± 6603  (3270-45508) | 3501 ± 1641  (1256-10453) |

**Table S2a.** Summary of the model selection procedure to evaluate the factors influencing nesting site selection by Bonelli’s eagles in southern Portugal at the 250-m buffer scale. Models are ranked by decreasing value of AICc. For each model we provide the variables included, the degrees of freedom (df) the log-likelihood (logLik), the Aikaike Information Criteria corrected for small sample sizes (AICc), the variation in AICc in relation to the top ranking model, and the Aikaike weight (*wi*). The 95% set of models used in model averaging are underlined in grey.

| **Model ID** | **Model parameters (250 m)** | **df** | **logLik** | **AICc** | **∆AICc** | ***wi*** |
| --- | --- | --- | --- | --- | --- | --- |
| 20 | PC1, PC2, D_NEST | 3 | -14.552 | 35.100 | 0.000 | 0.337 |
| 24 | PC1, PC2, PC3, D_NEST | 4 | -13.748 | 35.500 | 0.390 | 0.277 |
| 32 | PC1, PC2, PC3, PC4, D_NEST | 5 | -13.031 | 36.100 | 0.960 | 0.209 |
| 28 | PC1, PC2, PC4, D_NEST | 4 | -14.216 | 36.400 | 1.330 | 0.174 |
| 22 | PC1, PC3, D_NEST | 3 | -20.296 | 46.600 | 11.490 | 0.001 |
| 30 | PC1, PC3, PC4, D_NEST | 4 | -19.361 | 46.700 | 11.620 | 0.001 |
| 18 | PC1, D_NEST | 2 | -22.584 | 49.200 | 14.060 | 0.000 |
| 26 | PC1, PC4, D_NEST | 3 | -21.955 | 49.900 | 14.810 | 0.000 |
| 31 | PC2, PC3, PC4, D_NEST | 4 | -21.617 | 51.200 | 16.130 | 0.000 |
| 23 | PC2, PC3, D_NEST | 3 | -26.137 | 58.300 | 23.170 | 0.000 |
| 27 | PC2, PC4, D_NEST | 3 | -26.334 | 58.700 | 23.560 | 0.000 |
| 19 | PC2, D_NEST | 2 | -29.077 | 62.200 | 27.050 | 0.000 |
| 29 | PC3, PC4, D_NEST | 3 | -30.433 | 66.900 | 31.760 | 0.000 |
| 21 | PC3, D_NEST | 2 | -32.437 | 68.900 | 33.770 | 0.000 |
| 25 | PC4, D_NEST | 2 | -34.393 | 72.800 | 37.680 | 0.000 |
| 17 | D_NEST | 1 | -35.965 | 73.900 | 38.830 | 0.000 |
| 4 | PC1, PC2 | 2 | -73.981 | 152.000 | 116.860 | 0.000 |
| 8 | PC1, PC2, PC3 | 3 | -73.244 | 152.500 | 117.380 | 0.000 |
| 12 | PC1, PC2, PC4 | 3 | -73.965 | 153.900 | 118.830 | 0.000 |
| 16 | PC1, PC2, PC3, PC4 | 4 | -73.244 | 154.500 | 119.380 | 0.000 |
| 6 | PC1, PC3 | 2 | -85.285 | 174.600 | 139.470 | 0.000 |
| 14 | PC1, PC3, PC4 | 3 | -85.272 | 176.500 | 141.440 | 0.000 |
| 2 | PC1 | 1 | -88.176 | 178.400 | 143.250 | 0.000 |
| 10 | PC1, PC4 | 2 | -88.037 | 180.100 | 144.970 | 0.000 |
| 15 | PC2, PC3, PC4 | 3 | -133.999 | 274.000 | 238.890 | 0.000 |
| 7 | PC2, PC3 | 2 | -136.158 | 276.300 | 241.210 | 0.000 |
| 11 | PC2, PC4 | 2 | -141.582 | 287.200 | 252.060 | 0.000 |
| 3 | PC2 | 1 | -142.8 | 287.600 | 252.490 | 0.000 |
| 13 | PC3, PC4 | 2 | -151.359 | 306.700 | 271.610 | 0.000 |
| 5 | PC3 | 1 | -152.47 | 306.900 | 271.840 | 0.000 |
| 9 | PC4 | 1 | -160.183 | 322.400 | 287.260 | 0.000 |
| 1 | Null | 0 | NA | NA | NA | 0.000 |

**Table S2b.** Summary of the model selection procedure to evaluate the factors influencing nesting site selection by Bonelli’s eagles in southern Portugal at the 500-m buffer scale. Models are ranked by decreasing value of AICc. For each model we provide the variables included, the degrees of freedom (df) the log-likelihood (logLik), the Aikaike Information Criteria corrected for small sample sizes (AICc), the variation in AICc in relation to the top ranking model, and the Aikaike weight (*wi*). The 95% set of models used in model averaging are underlined in grey.

| **Model ID** | **Model parameters (500 m)** | **df** | **logLik** | **AICc** | **∆AICc** | ***Wi*** |
| --- | --- | --- | --- | --- | --- | --- |
| 32 | PC1, PC2, PC3, PC4, D_NEST | 5 | -15.534 | 41.100 | 0.000 | 0.338 |
| 24 | PC1, PC2, PC3, D_NEST | 4 | -16.661 | 41.300 | 0.250 | 0.298 |
| 20 | PC1, PC2, D_NEST | 3 | -18.116 | 42.200 | 1.160 | 0.189 |
| 28 | PC1, PC2, PC4, D_NEST | 4 | -17.530 | 43.100 | 1.990 | 0.125 |
| 22 | PC1, PC3, D_NEST | 3 | -20.293 | 46.600 | 5.520 | 0.021 |
| 30 | PC1, PC3, PC4, D_NEST | 4 | -19.394 | 46.800 | 5.720 | 0.019 |
| 18 | PC1, D_NEST | 2 | -22.794 | 49.600 | 8.520 | 0.005 |
| 26 | PC1, PC4, D_NEST | 3 | -22.275 | 50.600 | 9.480 | 0.003 |
| 31 | PC2, PC3, PC4, D_NEST | 4 | -21.487 | 51.000 | 9.910 | 0.002 |
| 23 | PC2, PC3, D_NEST | 3 | -25.518 | 57.000 | 15.970 | 0.000 |
| 27 | PC2, PC4, D_NEST | 3 | -28.026 | 62.100 | 20.980 | 0.000 |
| 19 | PC2, D_NEST | 2 | -30.501 | 65.000 | 23.930 | 0.000 |
| 29 | PC3, PC4, D_NEST | 3 | -29.923 | 65.800 | 24.780 | 0.000 |
| 21 | PC3, D_NEST | 2 | -31.857 | 67.700 | 26.640 | 0.000 |
| 25 | PC4, D_NEST | 2 | -34.273 | 72.500 | 31.480 | 0.000 |
| 17 | D_NEST | 1 | -35.965 | 73.900 | 32.860 | 0.000 |
| 4 | PC1, PC2 | 2 | -78.898 | 161.800 | 120.730 | 0.000 |
| 12 | PC1, PC2,PC4 | 3 | -78.768 | 163.500 | 122.470 | 0.000 |
| 8 | PC1, PC2, PC3 | 3 | -78.852 | 163.700 | 122.640 | 0.000 |
| 16 | PC1, PC2, PC3, PC4 | 4 | -78.721 | 165.400 | 124.370 | 0.000 |
| 2 | PC1 | 1 | -92.697 | 187.400 | 146.330 | 0.000 |
| 6 | PC1, PC3 | 2 | -92.239 | 188.500 | 147.410 | 0.000 |
| 10 | PC1, PC4 | 2 | -92.425 | 188.800 | 147.780 | 0.000 |
| 14 | PC1, PC3, PC4 | 3 | -91.974 | 189.900 | 148.880 | 0.000 |
| 15 | PC2, PC3, PC4 | 3 | -135.271 | 276.500 | 235.470 | 0.000 |
| 7 | PC2, PC3 | 2 | -136.345 | 276.700 | 235.620 | 0.000 |
| 3 | PC2 | 1 | -141.945 | 285.900 | 244.820 | 0.000 |
| 11 | PC2, PC4 | 2 | -141.148 | 286.300 | 245.230 | 0.000 |
| 5 | PC3 | 1 | -155.154 | 312.300 | 271.240 | 0.000 |
| 13 | PC3, PC4 | 2 | -154.828 | 313.700 | 272.590 | 0.000 |
| 9 | PC4 | 1 | -160.564 | 323.100 | 282.060 | 0.000 |
| 1 | Null | 0 | NA | NA | NA | 0.000 |

**Table S2c.** Summary of the model selection procedure to evaluate the factors influencing nesting site selection by Bonelli’s eagles in southern Portugal at the 1000-m buffer scale. Models are ranked by decreasing value of AICc. For each model we provide the variables included, the degrees of freedom (df) the log-likelihood (logLik), the Aikaike Information Criteria corrected for small sample sizes (AICc), the variation in AICc in relation to the top ranking model, and the Aikaike weight (*wi*). The 95% set of models used in model averaging are underlined in grey.

| **Model ID** | **Model parameters (1000 m)** | **df** | **logLik** | **AICc** | **∆AICc** | ***Wi*** |
| --- | --- | --- | --- | --- | --- | --- |
| 32 | PC1, PC2, PC3, PC4, D_NEST | 5 | -12.623 | 35.200 | 0.000 | 0.602 |
| 28 | PC1, PC2, PC4, D_NEST | 4 | -14.893 | 37.800 | 2.540 | 0.169 |
| 24 | PC1, PC2, PC3, D_NEST | 4 | -15.491 | 39.000 | 3.740 | 0.093 |
| 20 | PC1, PC2, D_NEST | 3 | -16.857 | 39.700 | 4.470 | 0.064 |
| 30 | PC1, PC3, PC4, D_NEST | 4 | -16.192 | 40.400 | 5.140 | 0.046 |
| 22 | PC1, PC3, D_NEST | 3 | -18.676 | 43.400 | 8.110 | 0.010 |
| 26 | PC1, PC4, D_NEST | 3 | -18.757 | 43.500 | 8.270 | 0.010 |
| 18 | PC1, D_NEST | 2 | -20.517 | 45.000 | 9.790 | 0.005 |
| 31 | PC2, PC3, PC4, D_NEST | 4 | -19.615 | 47.200 | 11.980 | 0.002 |
| 23 | PC2, PC3, D_NEST | 3 | -25.443 | 56.900 | 21.640 | 0.000 |
| 27 | PC2, PC4, D_NEST | 3 | -25.688 | 57.400 | 22.130 | 0.000 |
| 19 | PC2, D_NEST | 2 | -29.501 | 63.000 | 27.760 | 0.000 |
| 29 | PC3, PC4, D_NEST | 3 | -28.682 | 63.400 | 28.120 | 0.000 |
| 21 | PC3, D_NEST | 2 | -32.165 | 68.300 | 33.080 | 0.000 |
| 25 | PC4, D_NEST | 2 | -32.676 | 69.400 | 34.110 | 0.000 |
| 17 | D_NEST | 1 | -35.965 | 73.900 | 38.690 | 0.000 |
| 4 | PC1, PC2 | 2 | -76.684 | 157.400 | 122.120 | 0.000 |
| 8 | PC1, PC2, PC3 | 3 | -76.553 | 159.100 | 123.860 | 0.000 |
| 12 | PC1, PC2, PC4 | 3 | -76.597 | 159.200 | 123.950 | 0.000 |
| 16 | PC1, PC2, PC3, PC4 | 4 | -76.446 | 160.900 | 125.650 | 0.000 |
| 2 | PC1 | 1 | -89.391 | 180.800 | 145.540 | 0.000 |
| 6 | PC1, PC3 | 2 | -88.890 | 181.800 | 146.530 | 0.000 |
| 10 | PC1, PC4 | 2 | -89.390 | 182.800 | 147.530 | 0.000 |
| 14 | PC1, PC3, PC4 | 3 | -88.890 | 183.800 | 148.530 | 0.000 |
| 15 | PC2, PC3, PC4 | 3 | -130.406 | 266.800 | 231.570 | 0.000 |
| 7 | PC2, PC3 | 2 | -131.622 | 267.200 | 232.000 | 0.000 |
| 3 | PC2 | 1 | -135.918 | 273.800 | 238.590 | 0.000 |
| 11 | PC2, PC4 | 2 | -135.070 | 274.100 | 238.900 | 0.000 |
| 5 | PC3 | 1 | -154.512 | 311.000 | 275.780 | 0.000 |
| 13 | PC3, PC4 | 2 | -153.987 | 312.000 | 276.730 | 0.000 |
| 9 | PC4 | 1 | -160.420 | 322.800 | 287.600 | 0.000 |
| 1 | Null | 0 | NA | NA | NA | 0.000 |


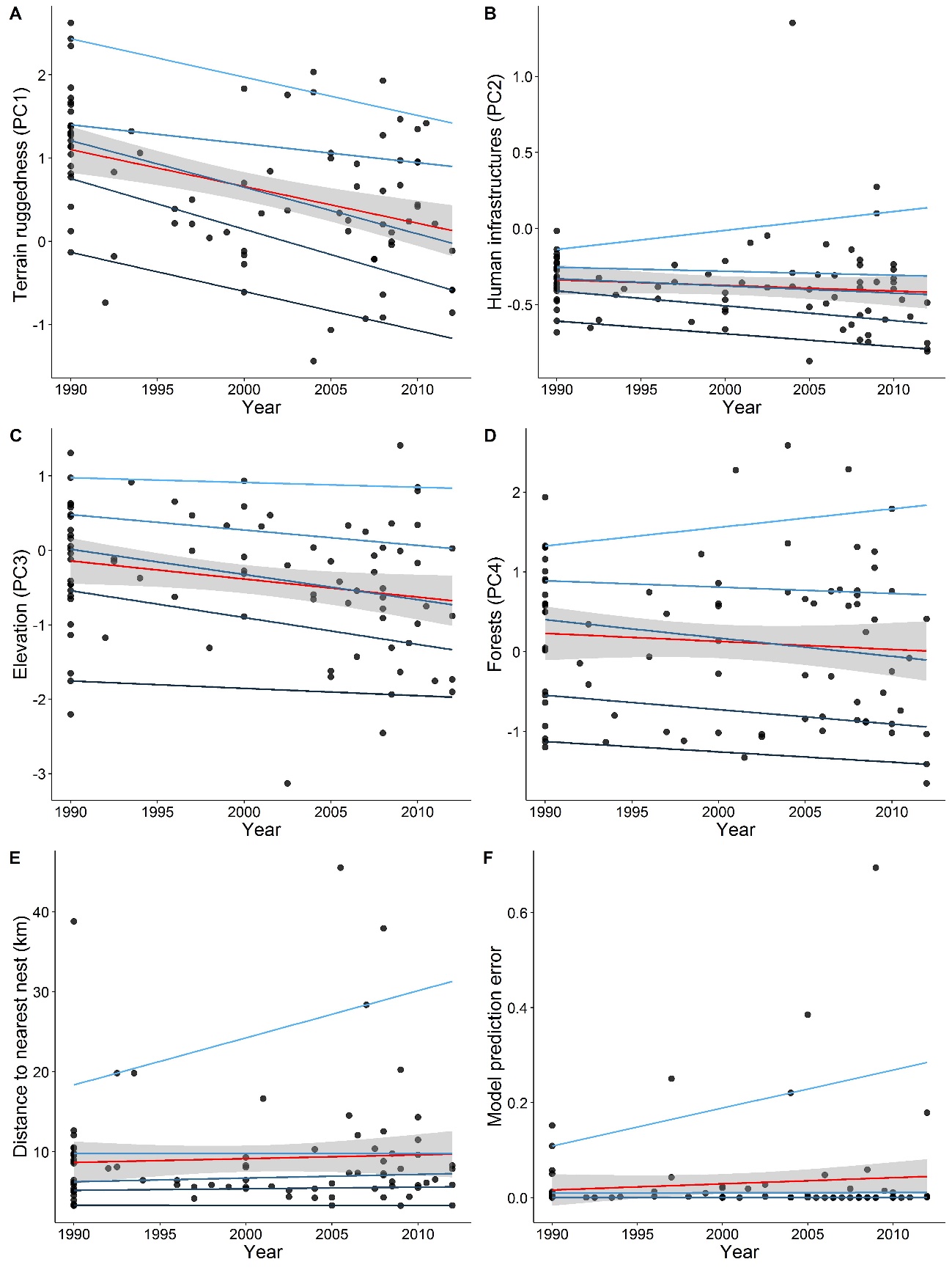


**Figure S1** – Scatterplots showing trends in habitat conditions around Bonelli’s eagle nests (250-m buffer) in relation to the year of territory establishment. Trends were estimated using ordinary least squares regression (red line, confidence intervals in grey) and quantile regression (light blue to dark blue lines), considering the habitat gradients extracted from a Principal Component Analysis (PC#), the distances to the nearest nest from a neighbouring territory, and the prediction error of the habitat model. The quantiles represented are 5% (dark blue), 25%, 50%, 75% and 95% (light blue).


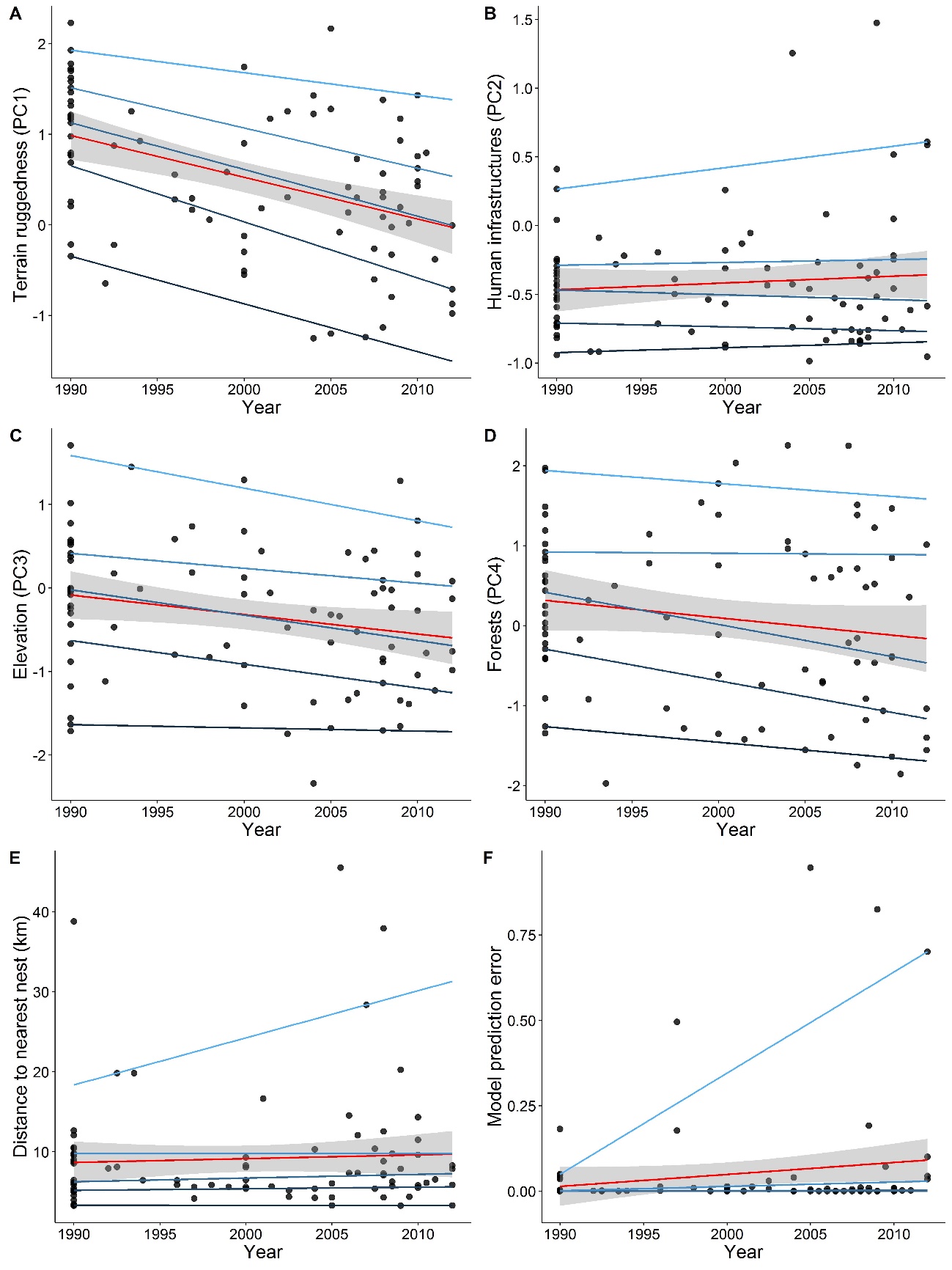


**Figure S2** – Scatterplots showing trends in habitat conditions around Bonelli’s eagle nests (1000-m buffer) in relation to the year of territory establishment. Trends were estimated using ordinary least squares regression (red line, confidence intervals in grey) and quantile regression (light blue to dark blue lines), considering the habitat gradients extracted from a Principal Component Analysis (PC#), the distances to the nearest nest from a neighbouring territory, and the prediction error of the habitat model. The quantiles represented are 5% (dark blue), 25%, 50%, 75% and 95% (light blue).

| 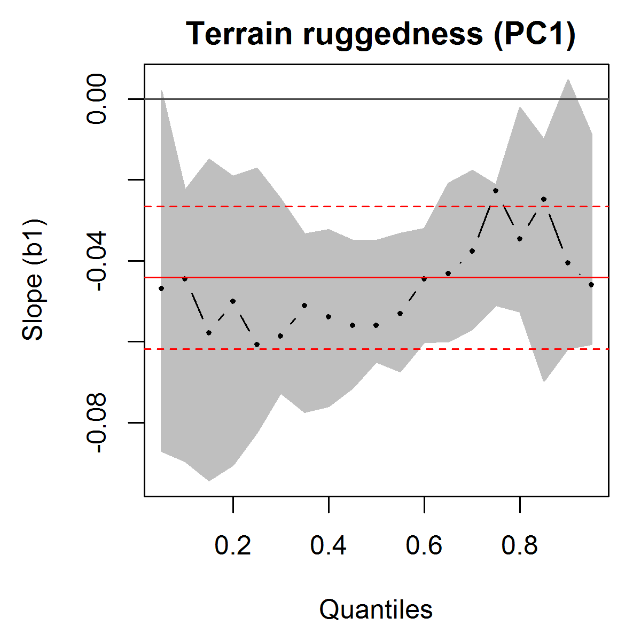 | 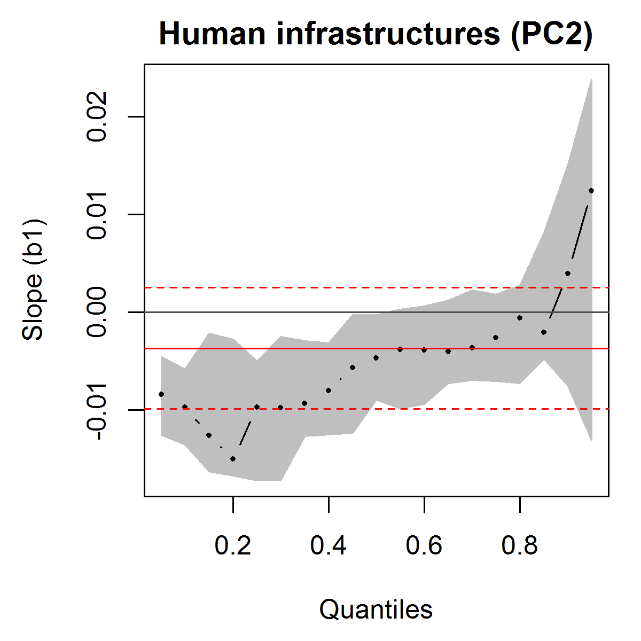 |
| --- | --- |
| 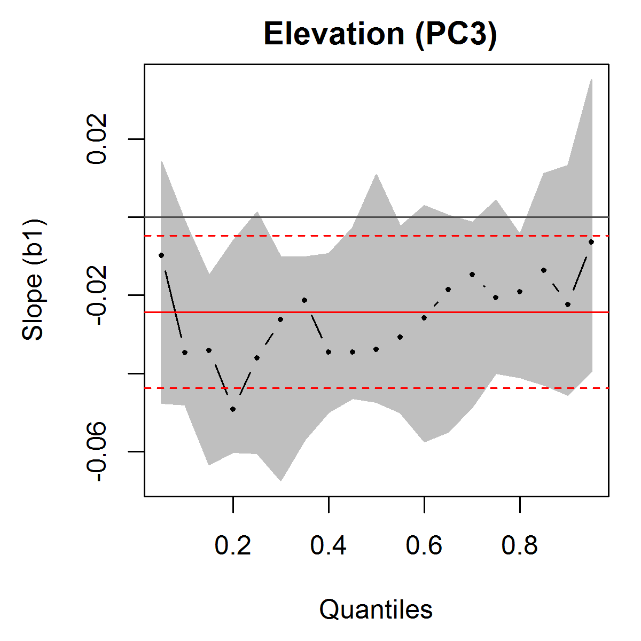 | 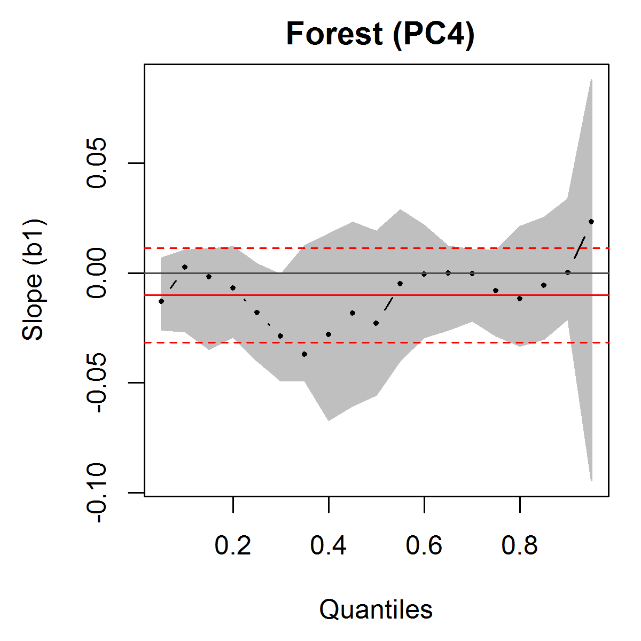 |
| 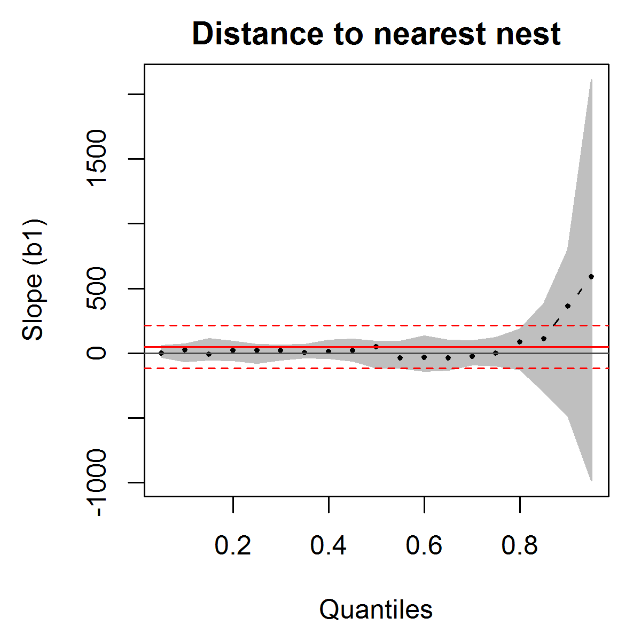 | 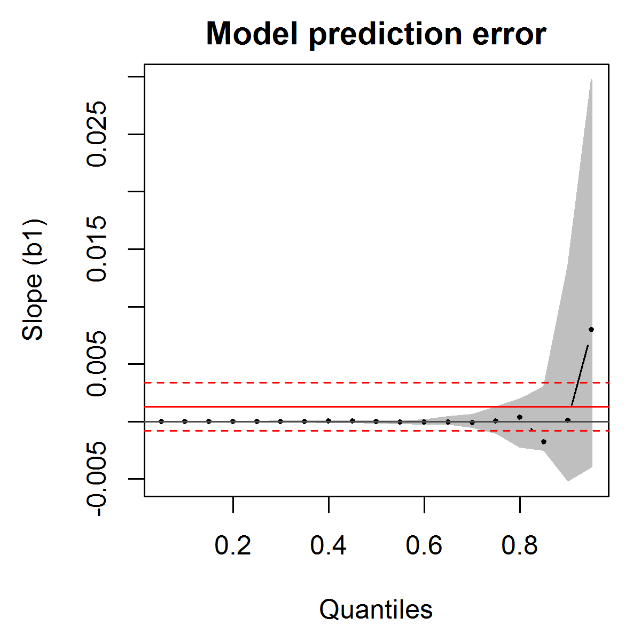 |

**Figure S3** – Estimated slopes (black circles) and its 90% confidence intervals (grey area) for quantile regression models (5% to 95%, at 5% increments) relating habitat conditions around Bonelli’s eagle nests (250-m) to the year of territory establishment. We also provide the slope (red line) and confidence bounds (hatched red line) for the slope estimated with ordinary least squares regression.

| 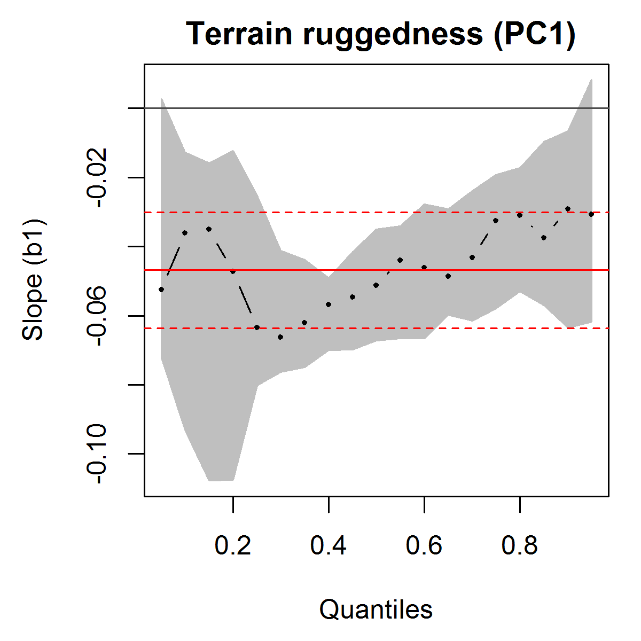 | 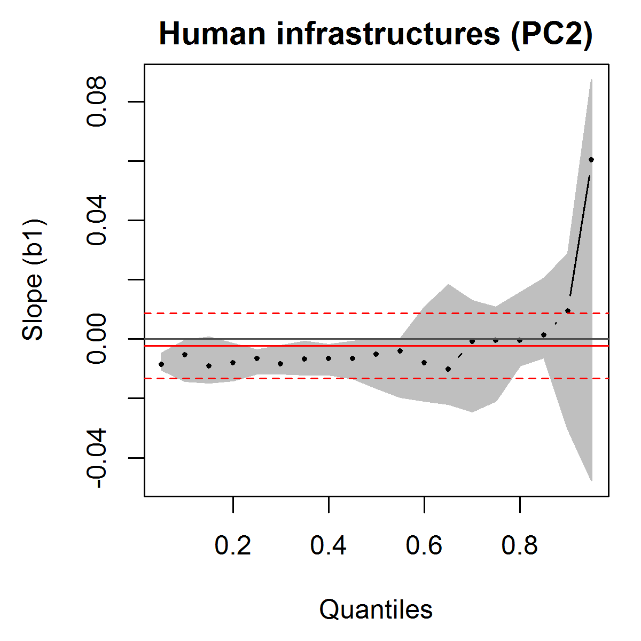 |
| --- | --- |
| 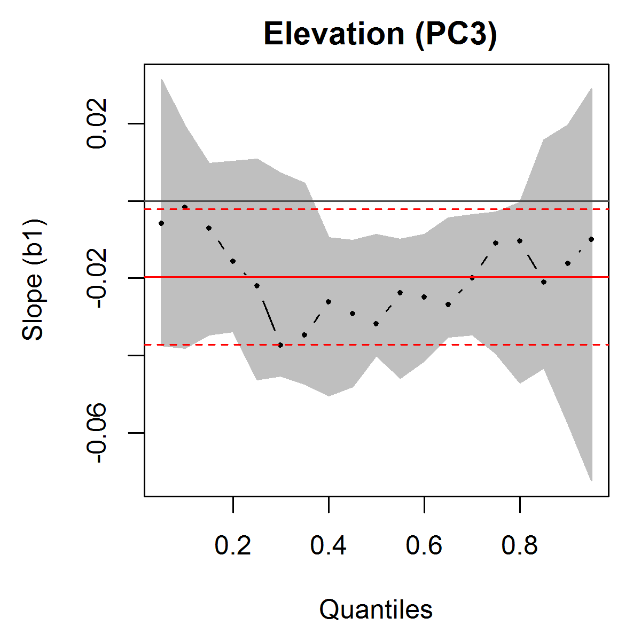 | 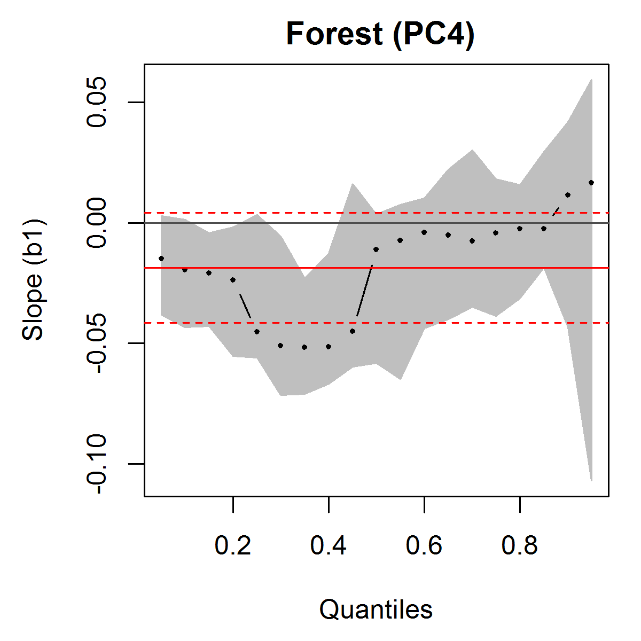 |
| 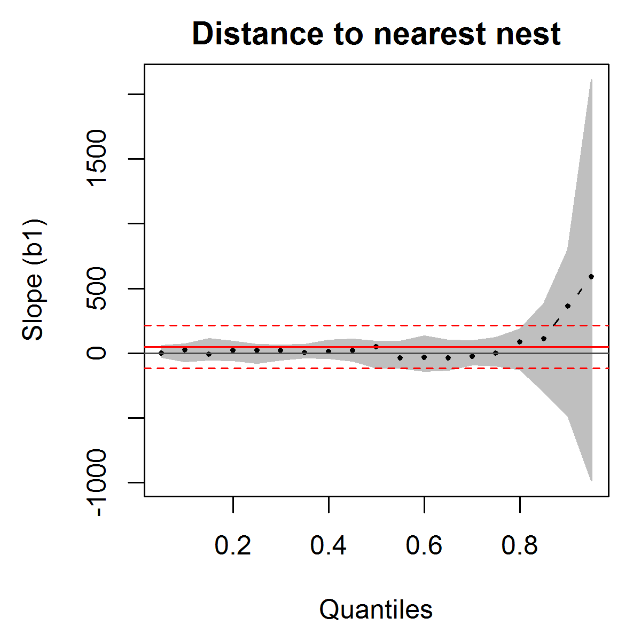 | 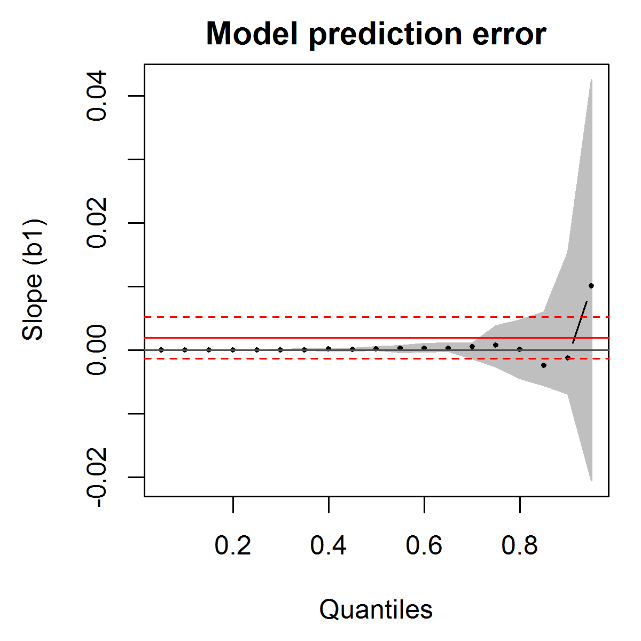 |

**Figure S4** – Estimated slopes (black circles) and its 90% confidence intervals (grey area) for quantile regression models (5% to 95%, at 5% increments) relating habitat conditions around Bonelli’s eagle nests (500-m) to the year of territory establishment. We also provide the slope (red line) and confidence bounds (hatched red line) for the slope estimated with ordinary least squares regression.

| 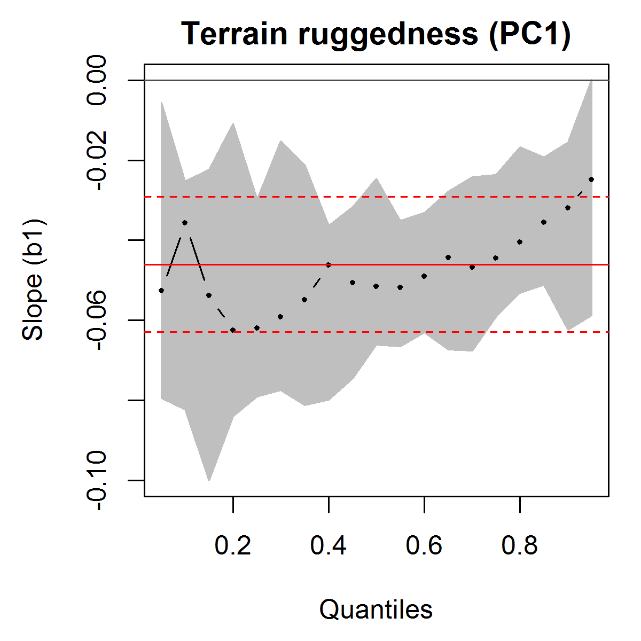 | 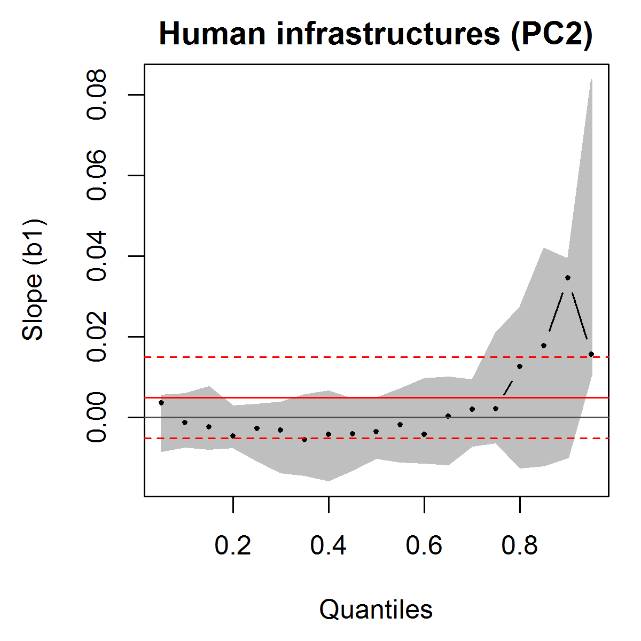 |
| --- | --- |
| 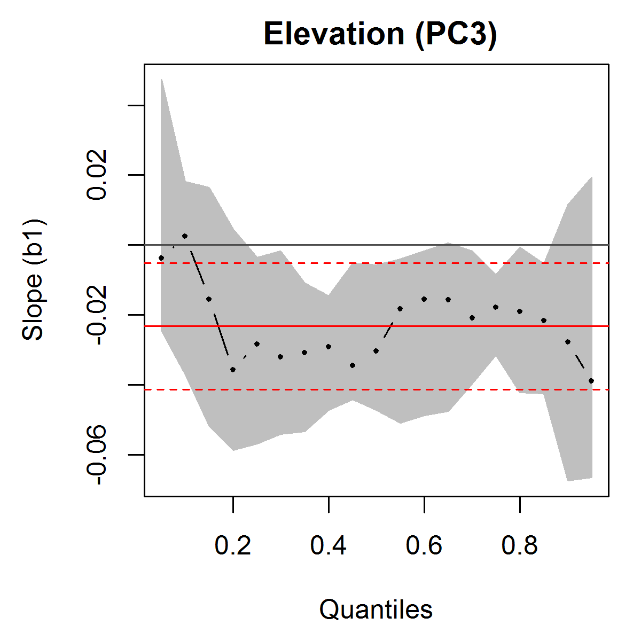 | 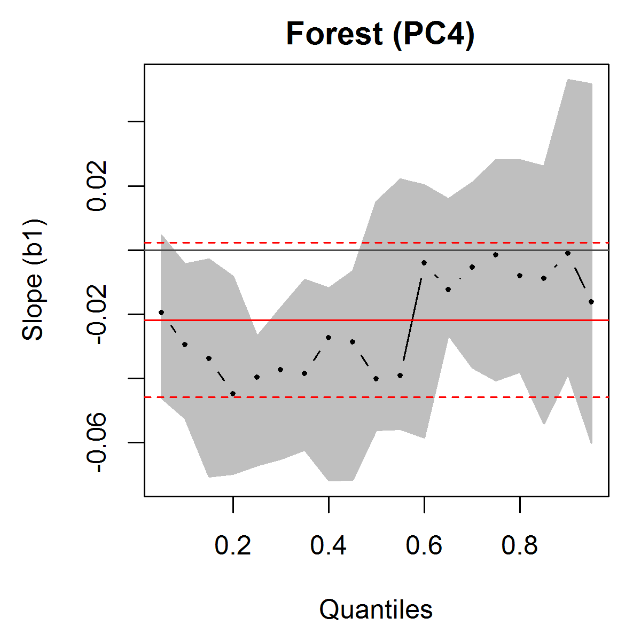 |
| 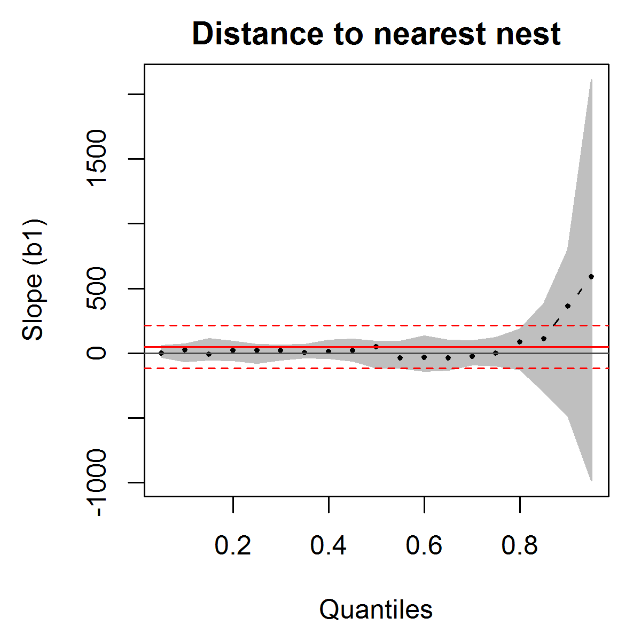 | 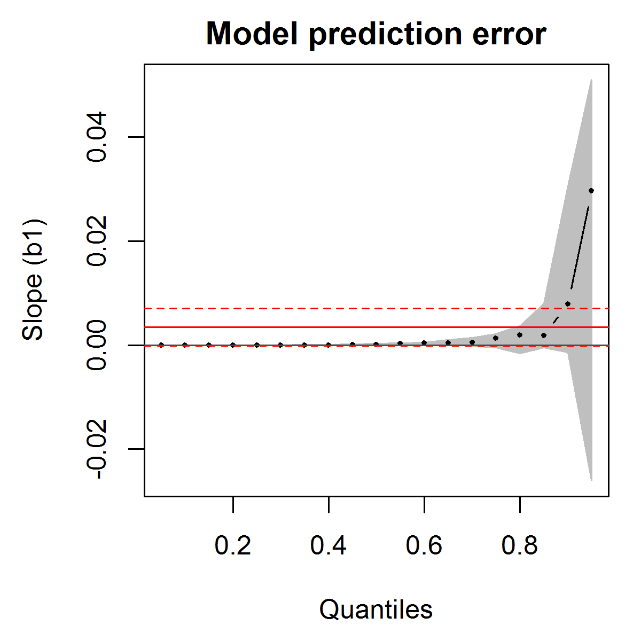 |

**Figure S5** – Estimated slopes (black circles) and its 90% confidence intervals (grey area) for quantile regression models (5% to 95%, at 5% increments) relating habitat conditions around Bonelli’s eagle nests (1000-m) to the year of territory establishment. We also provide the slope (red line) and confidence bounds (hatched red line) for the slope estimated with ordinary least squares regression.
